# Generation of Localized Surface Plasmon Resonance Using Hybrid Au–Ag Nanoparticle Arrays as a Sensor of Polychlorinated Biphenyls Detection

**DOI:** 10.3390/s16081241

**Published:** 2016-08-05

**Authors:** Jing Liu, Haoyuan Cai, Chaoyang Chen, Guangsong Yang, Cheng-Fu Yang

**Affiliations:** 1 School of Information Engineering, Jimei University, Xiamen 361021, China; jingliu@jmu.edu.cn (J.L.); xx002@jmu.edu.cn (C.C.); gsyang@jmu.edu.cn (G.Y.); 2 State Key Laboratory of Modern Optical Instrumentation, Department of Optical Engineering, Zhejiang University, Hangzhou 310027, China; haoyuancai1@gmail.com; 3 Department of Chemical and Materials Engineering, National University of Kaohsiung, No. 700, Kaohsiung University Rd., Nan-Tzu District, Kaohsiung 811, Taiwan

**Keywords:** hybrid Au–Ag periodic nanoparticle arrays, discrete dipole approximation (DDA), sensor, β-cyclodextrins (SH-β-CD), polychlorinated biphenyl

## Abstract

In this study, the hybrid Au–Ag hexagonal lattice of triangular and square lattice of quadrate periodic nanoparticle arrays (PNAs) were designed to investigate their extinction spectra of the localized surface plasmon resonances (LSPRs). First, their simulating extinction spectra were calculated by discrete dipole approximation (DDA) numerical method by changing the media refractive index. Simulation results showed that as the media refractive index was changed from 1.0 to 1.2, the maximum peak intensity of LSPRs spectra had no apparent change and the wavelength to reveal the maximum peak intensity of LSPRs spectra was shifted lower value. Polystyrene (PS) nanospheres with two differently arranged structures were used as the templates to deposit the hybrid Au–Ag hexagonal lattice of triangular and square lattice of quadrate periodic PNAs by evaporation method. The hybrid Au–Ag hexagonal lattice of triangular and square lattice of quadrate PNAs were grown on single crystal silicon (c-Si) substrates, and their measured extinction spectra were compared with the calculated results. Finally, the fabricated hexagonal lattices of triangular PNAs were investigated as a sensor of polychlorinated biphenyl solution (PCB-77) by observing the wavelength to reveal the maximum extinction efficiency (*λ*_max_). We show that the adhesion of β-cyclodextrins (SH-β-CD) on the hybrid Au–Ag hexagonal lattice of triangular PNAs could be used to increase the variation of *λ*_max_. We also demonstrate that the adhesion of SH-β-CD increases the sensitivity and detection effect of PCB-77 in hexagonal lattice of triangular PNAs.

## 1. Introduction

Surface plasmons are coherent delocalized electron oscillations that exist at the interface between any two materials where the real part of the dielectric function changes sign across the interface. If a metal film has two parallel boundaries, the surface plasmon waves will reflect, and the surface plasmons form the particular frequencies of standing waves on the metal nanoparticles with sizes smaller than the wavelength of light. The standing waves are called localized surface plasmon resonances (LSPRs). LSPRs are optical phenomena that can be generated as the optical light interacts with conductive metal or nanoparticles (NPs) with the size smaller than the incident wavelength of the used light. LSPRs are non-propagating excitations of the conduction electrons of metallic nanoparticles coupled to the electromagnetic field [1]. Since the first application of surface plasmon resonance (SPR) phenomenon for gas detection and biological sensor in 1982 [2], the SPR sensing technology has been widely used for the detection of biological and chemical analytes, environmental monitoring, and medical diagnostics [3,4] for the past two decades. The underlying phenomena exploited for nanobiophotonic sensing applications is based on the interaction of light and matters at nanoscale surfaces.

In the past, many different structures were investigated to obtain the characteristics of the localized surface plasmon resonance in a matrix. For example, Brolo and co-workers were the first group using arrays of sub-wavelength nanoholes in a gold film to monitor the binding of organic and biological molecules to a metallic surface [5]. The technique is based on the resonant surface plasmons transmission being enhanced through nanoholes in a collinear optical arrangement. Any change in the surface composition or geometry of the nanoholes is accompanied with a shift of wavelength of surface plasmon resonance. In addition, Steele and Brett used the Ag spheres and tilt nanowires, Si chevrons, and helical posts [6] to design the LSPRs devices, Huang et al. investigated nanotubes with matrix structure for the LSPRs devices [7]. Yamamichi et al. used colloidal gold as nanometric metal particles to study the design factors and the influence of interparticle spacing on sensing abilities [8].

When the nanoparticles are much smaller than the wavelength of light, the distribution of surface charge can be calculated using a simple electrostatic theory [9,10]. Exact solutions to Maxwell equations are known only for special geometries such as spheres, spheroids, or cylinders, so approximate methods are in general required. The basic idea of the discrete dipole approximation (DDA) was introduced in 1964 by DeVoe [11] who applied the DDA method to study the optical properties of aggregate moleculars. However, DDA algorithm employs no physical approximations and can produce accurate and enough results. Thus, DDA is a convenient method to compute light scattering and absorption properties of radiation from nanoparticles having arbitrary shapes and having periodic structures [12], including nanoparticle arrays. In addition, we can use the DDA method to calculate the extinction spectra and refractive index sensitivity (RIS) value in the nanoparticle arrays.

During fabrication of metal nanoparticle arrays, nanoparticle monomers with different shapes have been found to display a single dipole resonance peak, for example, sphere, cube, tetrahedron, octahedron, triangular plate, or the rectangular bars with different aspect ratios [12]. Noble metal nanoparticles exhibit rich LSPRs properties. Typical metals that support surface plasmons are silver (Ag) [13], gold (Au) [14], and copper (Cu) [15] single-layer thin films and hybrid Ag–Au [16] and hybrid Ni–Au [17] bi-layer thin films, and Cr can be used as the interlayer [13,16] to improve the adhesion effect of the nanoparticle arrays. The methods that have been investigated include electron-beam lithography (EBL) [18] and photolithography [19] methods, which can be used to fabricate the structures of nanoparticle arrays for the generation of the LSPRs. Nanosphere lithography (NSL) is a powerful, low-cost, and high-efficiency fabrication technique for the creation of nanoparticle arrays with controlled size, shape, and interparticle spacing with periodically and geometrically tunable nanostructure arrays [16,20].

In this study, NSL was used as the method to fabricate hybrid Au–Ag nanoparticle arrays for the generation of the localized surface plasmon resonances (LSPRs) and investigate their extinction spectra. Because the shape of the metallic nanoparticle arrays dictates the spectral signatures of the plasmon resonance, the abilities to change the parameters and to study the effect of parameters on the properties of LSPRs are important experimental challenges. For this reason, two different arrangements of polystyrene (PS) nanospheres were used as a deposition mask to deposit the hexagonal lattice of triangular and square lattice of quadrate periodic nanoparticle arrays (PNAs). First, the DDA method was used to find the better RIS and figure of merit (FOM) values, the periodic hybrid Au–Ag triangular nanoparticle array was systematically grown on the single crystal silicon. The most important reason for using hybrid Au–Ag nanoparticle is that the Au can inhibit Ag to happen the oxidation and sulfide. The adhesive ability of the hexagonally arranged triangular hybrid Au–Ag nanoparticle arrays to silicon substrate can be remarkably promoted by introducing a certain thickness of Cr interlayer. According to the theoretical calculations and experimental results, we could obtain a suitable Cr interlayer thickness of 8 nm [16]. Therefore, the Cr with thickness of 8 nm was deposited as the interlayer. In this study, we first explored the optical properties of the hybrid Au–Ag hexagonal lattice of triangular and square lattice of quadrate PNAs to the localized surface plasmon resonances (LSPRs) spectra.

A polychlorinated biphenyl (PCB) is a synthetic, organic chlorine compound derived from biphenyl, which is a molecule composed of two benzene rings. The toxicity of PCBs varies considerably among congeners. The coplanar PCBs, known as nonortho, are not substituted at the ring positions ortho to the other ring (for example, PCBs 77, 126, 169, etc.), and they tend to have dioxin-like properties and are generally among the most toxic congeners. Because PCBs are almost invariably found in complex mixtures, the concept of toxic equivalency factors (TEFs) has been developed to facilitate risk assessment and regulation, where more toxic PCB congeners are assigned higher TEF values on a scale from 0 to 1. Thus far, only few studies have investigated this despite the inhalation route of exposure and the continued presence of PCBs in indoor and ambient air. For LSPR detection, the analyzed molecules must be located near the metallic nanoparticles, implying that a special chemical moiety should be incorporated on the LSPR substrates first for enriching trace organic species. However, lipotropy and poor solubility in water are two important physical properties of all PCBs [21,22].

In the past, Fu et al. proposed a new device for special detection of PCB77 using aptamer-based SERS-microfluidic sensor [23]. They fabricated a highly sensitive SERS substrate with a structure of the Ag nanocrown by vacuum evaporation of Ag film on a patterned polydimethylsiloxane (PDMS). Then they used a surface-enhanced Raman scattering (SERS) for the measurement of PCB77. The Raman spectra have many peaks and it is difficult to confirm which peak can be used as the standard to judge the concentration of PCB77 [23,24]. Wang et al. showed that a sensitive fluorescent membrane of porous AAO template immobilized with phenyl isothiocyanate (PITC) (PITC@AAO) could detect 2,2’,4,5,5’-pentachlorinated biphenyl (PCB101) [25]. The fluorescence spectra in the PITC@AAO sensors also have many peaks and it is difficult to justify which peak can be used as the standard to judge the concentration of PCB101 [25]. Li et al. investigated ZnO-CuPc@porous-ZnO instead of bare porous-ZnO in the building of the PCBs-based sensor, but the CuPc chemisorptive bonding was difficult to calculate the variation for different concentration of PCBs [26].

In this study, we investigate periodic nanoparticle arrays as a new and high resolution sensor of polychlorinated biphenyl solution that can easily measure the variation of *λ*_max_ in the LSPRs spectra to find the concentration of PCB-77. β-CDs, whose dimension are 7 Å in width and 9 Å in depth, are a series of cyclic oligosaccharides with a hydrophilic exterior and a hydrophobic cavity and they are capable of binding the hydrophobic structures [27,28]. In the complex inclusion, β-CDs can be used as receptors to capture PCBs molecules in solution, which is approved by theoretical simulation [28,29]. For this reason, we exploit the binding properties of β-cyclodextrins (β-CDs) for special small organic molecules of PCBs. The localized surface plasmon resonances (LSPRs) can be used as a sensor by observing the variation of wavelength for the maximum extinction efficiency (*λ*_max_) of LSPRs spectra. Finally, we investigate the fabricated hexagonal lattices of triangular periodic nanoparticle arrays (PNAs) as a sensor of polychlorinated biphenyl solution (PCB-77 was used in this study) by observing the variation of *λ*_max_. We show that the synthesis and characterization of SH-β-CD can be used to increase the variation of *λ*_max_, which causes the increase of detection effect of PCB-77. For this reason, the fabricated hexagonal lattices of triangular PNAs were also dipped in the SH-β-CD solution to find the variation of *λ*_max_.

## 2. Model Construction and Simulation

**Figure 1:**
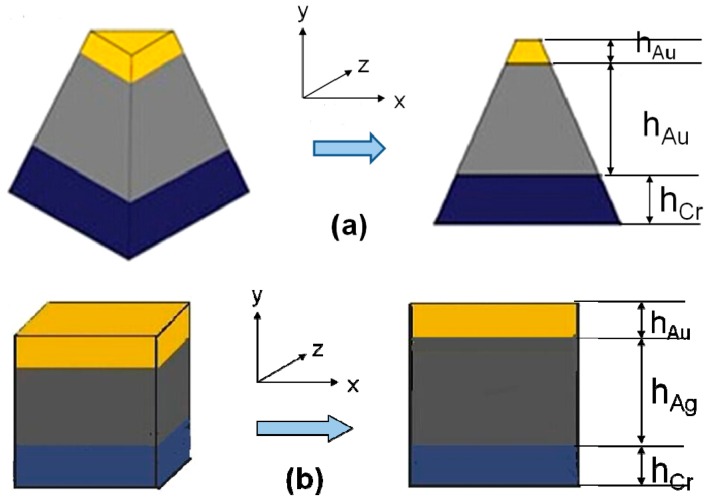
A monomer model of the hybrid Au–Ag nanoparticle arrays in 3D view and side cross-section view: (**a**) triangle; and (**b**) square.

The discrete dipole approximation (DDA), which can be used to model the experimentally measured extinction spectra, is an implementation of electrodynamic theory for calculating absorption and scattering by targets that have sizes smaller than or comparable to the wavelength of the incident radiation. In the past, we obtained a suitable Cr interlayer thickness according to the theoretical calculations and experimental results [16]. Figure 1 illustrates the schematic side view for the designed structure of the hybrid Au–Ag nanoparticle arrays. The thicknesses of Cr, Ag, and Au thin films are defined in Figure 1, and c-Si was used as the substrate. Corresponding schematic illustration of the hybrid nanoprism, the monomer models of the hybrid Au–Ag nanoparticle arrays were equilateral triangle (Figure 1a) and square (Figure 1b), and in-plane widths of each nanoparticles was 180 nm. The out-of-plane heights of the Cr and Ag nanoparticles under the Au layer were 60 nm and 35 nm, respectively, and the top Au nanoparticles was only 5 nm.

**Figure 2:**
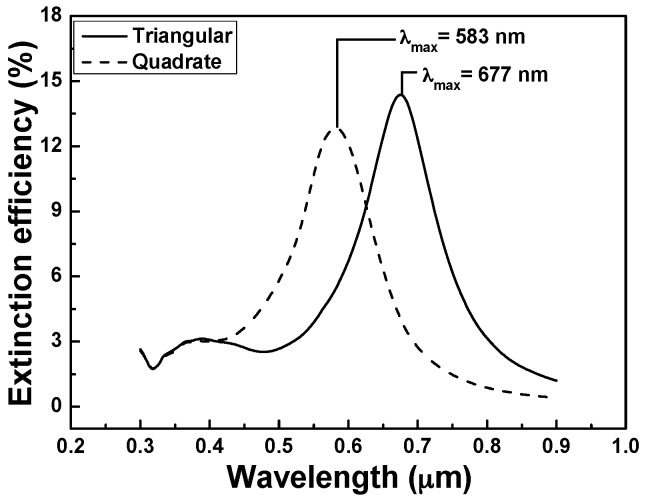
Extinction spectra of the hybrid Au–Ag nanoparticle arrays as a function of monomer shape.

Nanoscale researches for LSPRs applications are essentially focused on optics studies of nanostructures and signal enhancement using nanoparticles. Extinction coefficient is an important parameter that can be obtained from the extinction spectra. Extinction coefficient refers to several different measures in physics to describe the absorption and scattering of electromagnetic radiation of light in a medium. Any changes in the parameters of metal nanoparticle arrays will lead to the optical drift in extinction spectrum and thus influence the optical applications in practice. Simulated LSPR spectra of the hybrid Au–Ag nanoparticle arrays for monomers with the shapes of hexagonal lattice of triangular and square lattice of quadrate are presented in Figure 2. As Figure 2 shows, the *λ*_max_ of LSPRs spectra was 583 nm and 677 nm for the hexagonal lattice of triangular and square lattice of quadrate hybrid Au–Ag nanoparticle arrays, respectively. The maximum extinction efficiency (14.37% at 677 nm) of LSPRs for the hexagonal lattice of triangular hybrid Au–Ag nanoparticle arrays was large than that (12.83% at 583 nm) of LSPRs for the square lattice of quadrate hybrid Au–Ag nanoparticle arrays.

The full width at half maximum (FWHM) of the extinction coefficient spectra of the square lattice of quadrate hybrid Au–Ag nanoparticle arrays was 112 nm, which is smaller than that of the hexagonal lattice of triangular hybrid Au–Ag nanoparticle arrays (128 nm). This result suggests that the monome shape has apparent effect on the *λ*_max_ of LSPRs spectra. Silver nanoprism gives three absorbance peaks at around 330 nm, 420 nm, and 600–800 nm, which correspond to the out-of-plane quadrupole, in-plane quadrupole, and in-plane dipole plasmon resonance [30]. In Figure 2, the spectrum of hexagonal lattice of triangular nanoparticle arrays shows typical behavior for a prism in which there was a strong in-plane dipolar excitation at 677 nm and that of tetragonal nanoparticle arrays in which there was a strong in-plane dipolar excitation at 583 nm. The weaker in-plane quadrupole resonance, which is the bluest resonance and always at 335 nm, is believed to combine with out-of-plane quadrupole resonance to form a broader resonance from 335 nm to 450 nm for the spectrum of hexagonal lattice of triangular nanoparticle arrays and from 335 nm to 410 nm for the spectrum of the square lattice of quadrate nanoparticle arrays. The results in Figure 2 show that the calculated extinction spectrum was very sensitive to the size of nanoparticle arrays, because the wavelengths of the in-plane dipole resonance and out-of-plane quadrupole resonance increased with increasing edge length.

**Figure 3:**
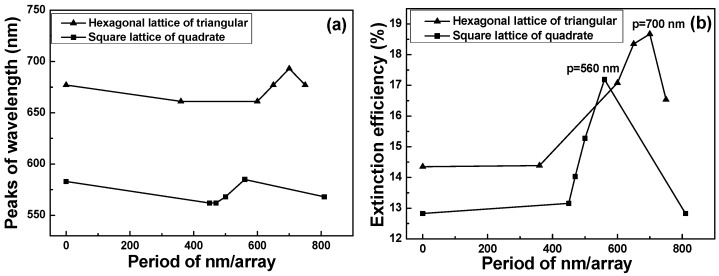
Relationships between: (**a**) the wavelength having the maximum extinction efficiency; and (**b**) the maximum extinction efficiency with the periods of the hybrid Au–Ag nanoparticle arrays as a function of monome shape.

The periodicity of the hexagonal lattice of triangular and square lattice of quadrate nanoparticle arrays also has important influence on the optical transmission properties, and we believe that will influence the properties of the extinction spectra. We calculated the effect of periodicity on the extinction spectra using the DDA algorithm aided design method, and the period was changed in the 0–810 nm range. Therefore, there are two important results observed from the simulated spectra. The relationships between the peak wavelength and the period of the hexagonal lattice of triangular and square lattice of quadrate nanoparticle arrays are shown in Figure 3a, where the first point stand for the single particle when the period was zero. The first important result is that the *λ*_max_ of LSPRs spectra has no apparently red shift with the increasing of periods. For the hexagonal lattice of triangular nanoparticle arrays, all *λ*_max_ of LSPRs are higher than those of the square lattice of quadrate nanoparticle arrays, independent of the period of nanoparticle arrays.

The relationships between the extinction efficiency and the periods of the hybrid Au–Ag hexagonal lattice of triangular and square lattice of quadrate PNAs are shown in Figure 3b. The second important result is that the peak heights of the extinction spectra first increase as the periods increase, reachin a maximum at 700 nm/array and 560 nm/array for hexagonal lattice of triangular and square lattice of quadrate nanoparticle arrays, respectively, and then decrease when further increasing the periods. This is easy to understandast coherence of the dipole sums of the localized surface plasmon resonances is expected to appear as a maximum for the period of a special nm/array. As Figure 3b shows, most peak heights of the extinction efficiency of the hexagonal lattice of triangular nanoparticle arrays are higher than those of the square lattice of quadrate nanoparticle arrays. As we know, nanoparticles assembled in the form of adsorbed layers have coupling in their electric fields that influences the LSPRs wavelength (or frequency) of the films, and the LSPRs wavelength depends mainly upon the nanoparticles size, array, shape, material property, and surrounding medium. Thus, the periods and sizes of the hybrid Au–Ag nanoparticle arrays will also influence the value of maximum extinction efficiency and the *λ*_max_ of LSPRs spectra. 

**Figure 4:**
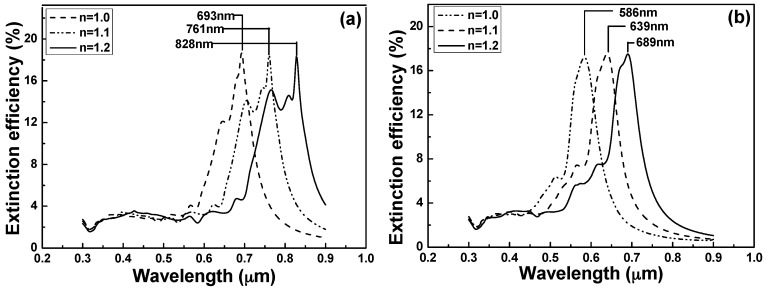
Extinction spectra in different media of nanoparticle arrays for different monome shape: (**a**) hexagonal lattice of triangular; and (**b**) square lattice of quadrate.

Their surface plasmon resonance peaks generally shift as the refractive index of the surrounding environment is changed. The dependence of their surface plasmon wavelengths on the surrounding refractive index is highly sensitive, which forms the basis of localized surface plasmon resonance spectroscopy [31,32]. To illustrate the effect of nanoparticle shape on the *λ*_max_ of LSPRs spectra dielectric sensitivity, the LSPR spectra of the two differently shaped nanoparticles were simulated in various solvent environments to investigate the effect of the substrates refractive indexes on the sensitivity of the hybrid nanostructure arrays. Thus, the extinction spectra of the effective refractive index of the medium surrounding the nanostructure arrays were calculated. The sensitivity of the LSPR to the refractive index of bulk external medium was simulated for each of the hybrid Au–Ag hexagonal lattice of triangular and square lattice of quadrate PNAs. As the PNAs were in hexagonal lattice of triangular structure and the medium refractive index was increased from 1.0 to 1.2, the FWHM value and peak value of LSPRs spectra had no apparent changes, the *λ*_max_ of LSPRs spectra was shifted from 828 nm to 693 nm. However, as Figure 4b shows, as the PNAs were in square lattice of quadrate structure and the medium refractive index was increased from 1.0 to 1.2, the FWHM value and peak value of LSPRs spectra had no apparent changes, the *λ*_max_ of LSPRs spectra was shifted from 689 nm to 586 nm.

**Figure 5:**
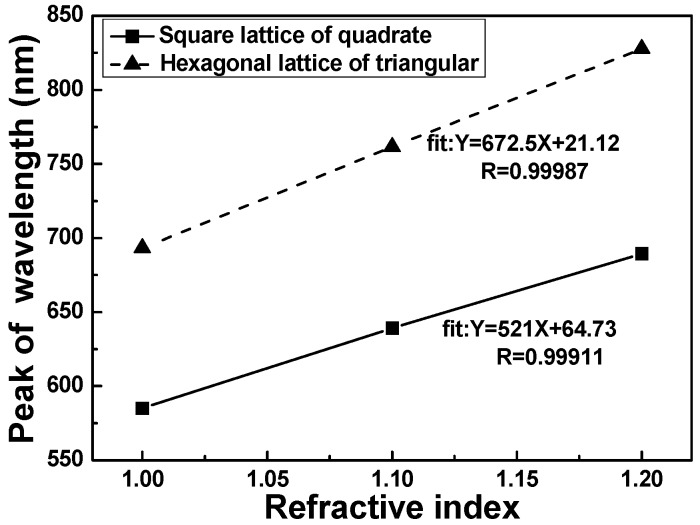
Refractive index sensitivity curves of nanoparticle arrays for different monome shape.

The results shown in Figure 4a,b suggest that the wavelength for peak value had a blue shift when the medium refractive index *n* increased. The RIS value is defined as m = ∆*λ/*∆*n* [5], where ∆*λ* denotes the change in peak value of the extinction spectrum and ∆*n* denotes the change in medium’s refractive index. Figure 5 shows the relationships between the wavelengths for peak value and the medium refractive index, which could be used to investigate the RIS values. As the PNAs were in hexagonal lattice of triangular structure, the RIS value of 673 nm/RIU (refractive index unit) was obtained in the hybrid nanostructure arrays; As the PNAs were in square lattice of quadrate structure, the RIS value of 521 nm/RIU was obtained in the hybrid nanostructure arrays, respectively. The FOM value for a metal nanostructure is defined as the ratio of RIS/FWHM, and the FOM values were 6.21 and 6.04, as the periodic nanoparticle arrays were in hexagonal lattice of triangular and square lattice of quadrate structures (Table 1). Those results prove that in the structure of PNAs, the change for the sensitivity of the LSPR *λ_max_* in the local dielectric environment is determined by the difference in nanoparticle geometry.

For nanoparticles that have sizes much smaller than the wavelength of the absorbing light, only the dipole term is assumed to contribute to the absorption. For silver nanoparticles, the dipole is about 3 nm and 5 nm for p-polarization and s-polarization, respectively [33]. In the quasi-static regime, the extinction coefficient k for N particles of volume V is then given by the following equation [34]:

(1) $$k=\frac{18\pi NV\varepsilon_{m}^{3/2}}{\lambda }\frac{\varepsilon_{2}}{{\left[\varepsilon_{1}+2\varepsilon_{m}\right]}^{2}+\varepsilon_{2}^{2}}$$

where λ is wavelength of the absorbing radiation; *ε*_m_ is the dielectric constant of the surrounding medium and it is assumed to be frequency independent; *ε*_1_ and *ε*_2_ are the real and imaginary parts of the material dielectric function, respectively; and *ε* (ω) = *ε*_1_ (ω) + *iε*_2_ (ω), where ω is the angular frequency of the light. The plasmon absorbance ratio *A* of a colloidal solution containing *N* particles in an optical cell with a path length *L* is *A* = (k/ln 10)*L*. The resonance condition for the plasmon absorption is roughly fulfilled when *ε*_1_ (ω) = −2*ε*_m_ if *ε*_2_ is small or weakly dependent on ω. The plasmon bandwidth mainly depends on *ε*_2_ (ω). According to Equation (1), the plasmon absorption is independent of the size of the fabricated nanoparticles within the dipole approximation. However, the simulation result of a size effect on the surface plasmon absorption was observed as the plasmon bandwidth increased with decreasing particle size. As the results in Figure 3a,b are compared, in fact the bandwidth of the extinction spectra is inversely proportional to the radius *r* of the particle for sizes smaller than about 20 nm. For simple hybrid Au–Ag nanoparticles, strong light absorbing and scattering occur at plasmon resonance frequency and high local fields are excited around the nano-particles [35]. However, in a system consisting of uniformly distributed hybrid Au–Ag PNAs, the resonant condition is modified for particle-particle interaction. The simulation results in Figure 3b show that as the size of nanoparticles is smaller, the maximum extinction efficiency will be higher.

## 3. Experimental

### 3.1. Materials

Beta-cyclodextrins (β-CDs, a white powder) and PCB-77 were purchased from Aladdin Chemical Co. Ltd. Toluene, acetone, carbinol, acetic acid, and ethanol were purchased from Lvyin Chemical Co. Ltd. Silver (99.9%) was purchased from Keda Materials Co. Ltd. The polystyrene (PS) nanospheres with a mean diameter of 360 nm and a concentration of 10 wt % in solution were purchased from Suzhou Nano-Micro Bio-Tech Co. Ltd. Deionized water was used throughout the experiments.

### 3.2. Synthesis of Mono-6-Thio-β-Cyclodextrin

The synthesis and characterization of β-CD (SH-β-CD) were same as reported in the literature [28,29,31]. A mixture of 2 g β-CDtos and 2 g thiourea in 100 mL 80% methanol–water (*v*/*v*) was heated at reflux for 2 days and the solvent of the reaction mixture was removed in vacuum. Subsequently, the white solid was added to 30 mL methanol and stirred for 1 h. After filtration, the residue was dissolved with a 10 wt % 69 mL NaOH solution and stirred at 50 °C for 5 h. When the pH value of the reaction mixture was adjusted to 2 with 10 wt % HCl, the pale yellow solution was formed. Then, 5 mL trichloroethylene was added to the solution and stirred overnight. The resulting white precipitate was recovered by suction filtration and washed with water. To confirm the presence of the group in the product, NaNO_2_ and HCl were added into the as-prepared solution. The color of the solution changed to red, which indicated the existence of the group in the product. After recrystallization, the white solid material was obtained. ^1^H NMR (400 MHz, (CD_3_)_2_SO, 25 °C, TMS, δ): d = 2.03 (m, SH), 2.50–3.20 (m, 2H), 3.26–3.47 (m, overlapping with HDO), 3.56–3.76 (m, 28H), 4.38–4.52 (m, 6H), 4.83–4.91 (br d, 7H), 5.59–5.84 (m, 14H) ppm. Anal. Calcd. For C_42_H_70_O_34_S·7H_2_O: C, 39.50; H, 6.63; S, 2.51. Found: C 39.23; H 7.02; S 2.38.

### 3.3. Fabrication of Nanostructures

When the results shown in Figure 2 and Figure 3a,b are compared, they suggest some important results. As the period of the hexagonal lattice of triangular nanoparticle arrays is 700 nm/array, it has the most matched condition with the resonant wavelength 677 nm of the single hybrid Au–Ag hexagonal lattice of triangular PNAs and the LSPRs has the maximum extinction efficiency. Similarly, as the period of the square lattice of quadrate nanoparticle arrays is 560 nm/array, it has the most matched condition with the resonant wavelength 583 nm of the single hybrid Au–Ag square lattice of quadrate film and the LSPRs has the maximum extinction efficiency. In order to find the effect of structure on the property of nanoparticle arrays, we used 700 nm/array and 560 nm/array as the templates to deposit the hybrid Au–Ag hexagonal lattice of triangular and square lattice of quadrate PNAs by evaporation method.

**Figure 6:**
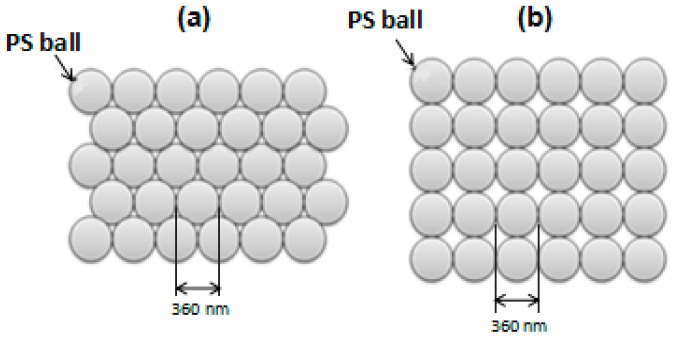
Two different arrangements of polystyrene (PS) nanospheres for the generations of: (**a**) hexagonal lattice of triangular; and (**b**) square lattice of quadrate Ag–Au hybrid nanoparticles arrays.

First, the c-Si wafers were used as substrates to study the effect of refractive index on the change of the LSPR peak value and optical property. NSL process began with the self-assembly of size-monodisperse nanospheres into a two-dimensional (2D) colloidal crystal and the nano-scale polystyrene (PS) balls were used as the nanospheres. In this study, the drop-coating method was used to construct the polystyrene (PS) nanospheres template with for different hybrid Au–Ag PNAs. Figure 6 illustrates the schematic top view for the fabrication structure of the PS arrays. As the solvent was evaporated at 45 °C, capillary forces would draw the PS nanospheres in the solution to diffuse freedom with the lowest free energy [36]. Then the nanospheres thereby arranged themselves into a hexagonally close-packed pattern on the substrate, (Figure 6a). As the evaporated temperature was 80 °C, the solvent of the PS nanospheres solution on the substrates would be dried in a fast speed and a non-stable state. Because the solution evaporated quickly, the PS nanospheres were kept in the unbalanced kinetic state [37]. This condition suggests that the PS nanospheres would keep in the metastable state, and then the nanospheres thereby arranged themselves into a tetragonal close-packed patterns on the substrate (Figure 6b). The PS nanospheres with an average diameter of 360 nm and a concentration of 10 wt % in solution were purchased from Suzhou Nano-Micro Bio-Tech Co. Ltd., Suzhou, China. The details for the preparation of the PS nanospheres were revealed in reference [13]. The deposition of Cr, Ag, and Au (all three metals with 3N purity) was performed in a self-built thermal evaporator at a pressure of 5.0 × 10^−4^ Pa. The glass substrates were rotated at a speed of 16.5 rpm during the deposition process.

The power for heating-up of the source materials was carefully increased in order to achieve homogeneous deposition. The deposition rate was about 4.0 nm/s for Cr thin film and the deposition rates were about 2.5 nm/s for both Au and Ag thin films. The thicknesses of the deposition thin films were monitored using a Dektak 3 Series surface profiler to achieve an identical depth for a low reflectance. Following the self-assembly process of the nanospheres mask, Cr, Ag, and Au thin films were deposited onto the PS nanosphere-coated substrates by thermal evaporation. The thicknesses of Cr (*h_cr_*), Ag (*h_Ag_*), and Au (*h_Au_*) were 8 nm, 35 nm, and 5 nm, respectively [38], by controlling their deposition time. After depositions of Cr, Ag, and Au thin films, the PS spheres were lifted off by immersing in absolute ethanol for about 5 s. The PS spheres were also removed by sonication (B3500S-MT, Branson, 140 W, 42 kHz) in absolute ethanol to examine the adhesive ability of the hybrid Au–Ag nanoparticles on different substrates. The achieved PS mask and the structures of the achieved hybrid nanoparticle arrays on different substrates were characterized by scanning electron microscope (LEO-1530, Leo Electron Microscopy, New York, NY, USA). Ultraviolet visible (UVvis) spectra were obtained on a Varian Cary 5000UV-Vis-NIR spectrophotometer.

### 3.4. Functionalization of the Nanosensor

**Figure 7:**
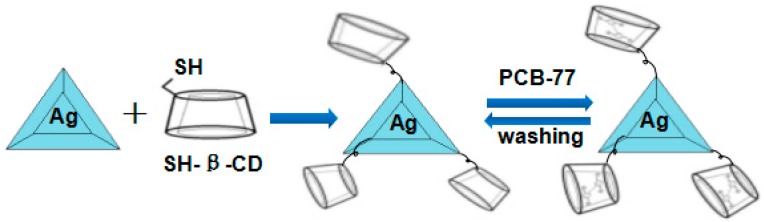
The modification process of SH-β-CDs and PCB-77 on the surface of the nanoparticle array.

In order to confirm the sensing effect of the polychlorinated biphenyl solution (PCB-77), the prepared LSPRs sensors with the structure of the hybrid Au–Ag hexagonal lattice of triangular PNAs were dipped into SH-β-CD solution for 2 h. We believed that he SH-β-CD would attach onto the surface of the hexagonal lattice of triangular PNAs through the adsorption of hydrosulfide groups. The hybrid Au–Ag hexagonal lattice of triangular PNAs having un-modified or modified SH-β-CD were used to measure the shift of LSPR *λ*_max_, which could be used as an index for sensing the concentration of PCB-77. Those results could be used to prove that attachment of SH-β-CD would improve the sensitivity of detecting PCB-77. The PCB-77 was dissolved in ethanol and diluted to the desired concentration with deionized water, then the mutterlauge of the hybrid Au–Ag hexagonal lattice of triangular PNAs was dipped. The sample was exposed to PCB-77 solution with different concentrations of 1 × 10^−5^ g/mL~1 × 10^−8^ g/mL. In order to check the repeatability of the sensing effect of the fabricated hybrid Au–Ag hexagonal lattice of triangular PNAs, the modified substrates were dipped into the solution of methanol and acetic acid to wash out PCB-77 and then dipped into PCB-77 solution again. Each hybrid Au–Ag hexagonal lattice of triangular PNA was repeated at least five times to confirm the repeatability of the sensing effect. The LSPRs’ absorption spectra were measured in each step, and the measured results would be compared in this study. The modification and functionalization process is shown in Figure 7.

## 4. Results and Discussion

**Figure 8:**
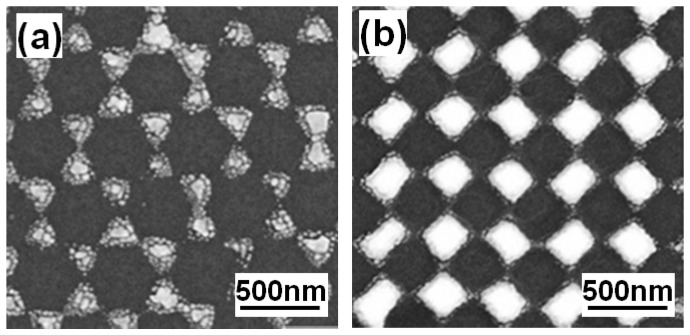
Surface morphologies of the fabricated hybrid Au–Ag: (**a**) hexagonal lattice of triangular; and (**b**) square lattice of quadrate periodic nanoparticle arrays.

SEM surface morphologies of the fabricated hybrid Au–Ag hexagonal lattice of triangular and square lattice of quadrate PNAs on a c-Si substrate are shown in Figure 8. The deposited hybrid Au–Ag hexagonal lattice of triangular nanoparticle arrays (Figure 8a) exhibited a hexagonally arranged disc structure rather than triangular structure and had a sharp angle in the triangular structure. The deposited hybrid Au–Ag square lattice of quadrate PNAs (Figure 8b) exhibited a tetragonally arranged disc structure. They were regular and well-defined as independent of the substrates, and no tiny cracks were observed in the structures. Those results suggest that the cohesive force between the PS nanospheres and the c-Si substrates seemed to be strong enough. Therefore, the results shown in Figure 8 suggest that different arrangements of PS nanospheres allow obtaining different PNAs.

**Figure 9:**
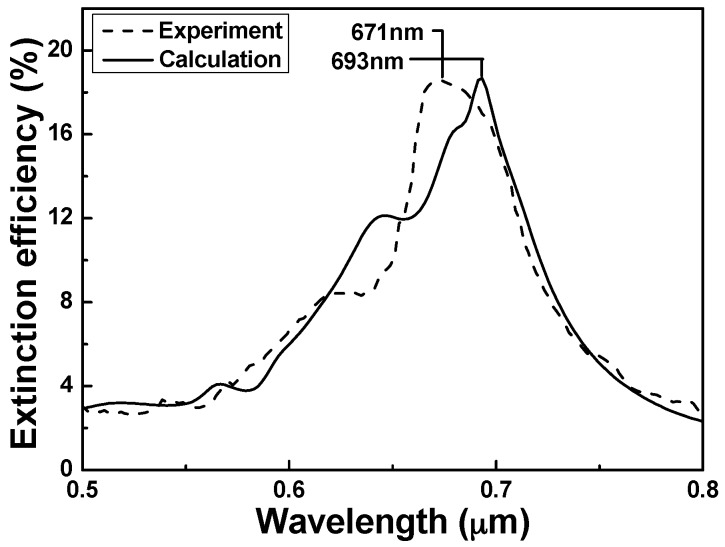
Localized surface plasmon resonance (LSPR) spectra comparing the simulation result and the measured response of hexagonal lattice of triangular periodic nanoparticle arrays.

**Figure 10:**
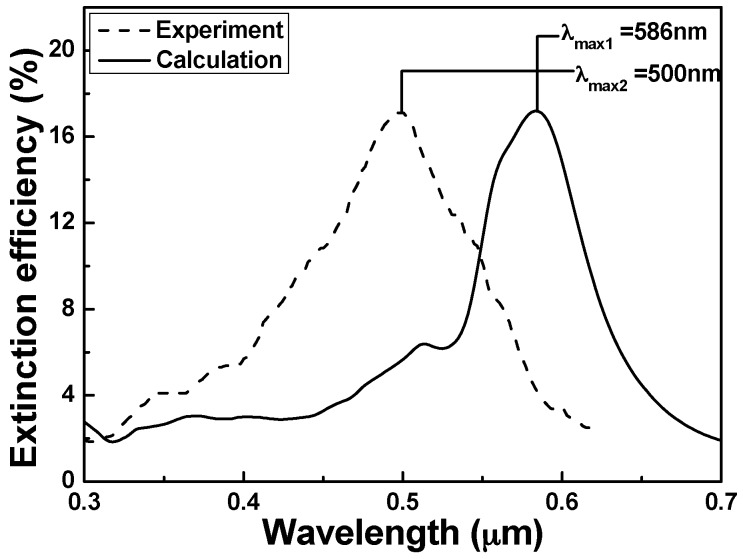
LSPR spectra comparing the simulation result and the measured response of square lattice of quadrate periodic nanoparticle arrays.

To investigate the applications of the hybrid Au–Ag PNAs as chemical-sensing platforms, the *λ*_max_ of LSPRs spectra responses to the extinction efficiencies of the representative hybrid PNAs in Figure 4 are detected with the incidence wavelength ranging from 500 nm to 800 nm. Figure 9 and Figure 10 compare the measured extinction spectra of the fabricated hybrid Au–Ag square lattice of quadrate and hexagonal lattice of triangular PNAs with those of the simulated results (from DDA numerical method) extrapolated from the data in Figure 4a,b, respectively. For the results of the DDA calculation, the *λ*_max_ of LSPRs spectra was 693 nm for the hexagonal lattice of triangular PNAs and was 586 nm for the square lattice of quadrate PNAs, whereas, for the experimental results, the *λ*_max_ of LSPRs spectra were 672 nm for the hexagonal lattice of triangular PNAs and 500 nm for the square lattice of quadrate PNAs.

Figure 9 and Figure 10 prove that the experimental results are generally in agreement with the calculated results. However, our DDA calculation and the experimental results show that the structure is an important parameter in determining the LSPRs extinction spectra, including the FWHM values and the *λ*_max_ of LSPRs. Figure 9 and Figure 10 suggest that as the sizes of nanoparticles are smaller, the FWHM value is smaller and the *λ*_max_ of LSPRs spectra is higher. The major difference between the calculation and experiment values was that the experimental peaks had the blue shifts of 22 nm and 86 nm for hexagonal lattice of triangular and square lattice of quadrate PNAs, respectively. In the case of the hexagonal lattice of triangular PNAs, the sensitivity is more similar to the simulated values than that of square lattice of quadrate PNAs. Thus, those results suggest that all of the chemosensing and biosensing techniques can be well developed by directly using the hexagonal lattice of triangular PNAs. The difference between the calculation and experiment results of the square lattice of quadrate PNAs was larger than that of the hexagonal lattice of triangular PNAs. From the SEM observation shows in Figure 8, the hybrid Au–Ag hexagonal lattice of triangular PNAs having the better uniformity is believed to be the major reason.

**Figure 11:**
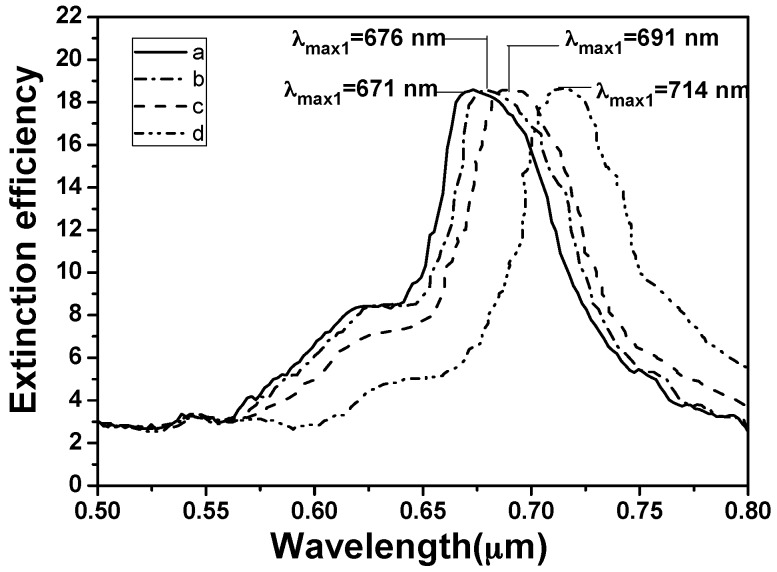
LSPR spectra of each step: (**a**) the hybrid Au–Ag hexagonal lattice of triangular PNAs; (**b**) the hybrid Au–Ag hexagonal lattice of triangular PNAs modified with the modification of SH-β-CD without dipping PCB-77; and the hybrid Au–Ag hexagonal lattice of triangular PNAs (**c**) without modification of SH-β-CD and (**d**) with the modification of SH-β-CD to measure the PCB-77 of 1 × 10^−5^ g/mL.

The absorption spectra of the hybrid Au–Ag hexagonal lattice of triangular PNAs, the hybrid Au–Ag hexagonal lattice of triangular PNAs modified with the SH-β-CD without dipping PCB-77, the hybrid Au–Ag hexagonal lattice of triangular PNAs dipped in PCB-77 alone and un-modified with SH-β-CD, and the hybrid Au–Ag hexagonal lattice of triangular PNAs modified with SH-β-CD (1 mM) and dipped in PCB-77 (1 × 10^−5^ g/mL, after chemical modification) are shown in Figure 11. The LSPR *λ*_max_ of the hybrid Au–Ag hexagonal lattice of triangular PNAs was revealed at 671 nm and the LSPR *λ*_max_ of the hybrid Au–Ag hexagonal lattice of triangular PNAs modified with the modification of SH-β-CD without dipping PCB-77 was revealed at 676 nm. As the hybrid Au–Ag hexagonal lattice of triangular PNAs without the modification of SH-β-CD were used to measure the PCB-77 of 1 × 10^−5^ g/mL, the LSPR *λ*_max_ was shifted to 691 nm. The results in Figure 11 show that the hybrid Au–Ag triangle hexagonal PNAs without modification of SH-β-CD and with the modification of SH-β-CD to measure the PCB-77 of 1 × 10^−5^ g/mL have different shift values of the LSPR *λ*_max_. Those results suggest that as SH-β-CD was used to modify the hybrid Au–Ag hexagonal lattice of triangular PNAs, the LSPR *λ*_max_ will have different result, which is caused by the different effective refractive index of the medium (SH-β-CD) surrounding the nanostructure arrays. The hybrid Au–Ag hexagonal lattice of triangular PNAs without the modification of SH-β-CD the sensing of PCB-77 of 1 × 10^−5^ g/mL has a 20 nm red shift from the original spectrum. The hybrid Au–Ag hexagonal lattice of triangular PNAs with the modification of SH-β-CD was used to sense the PCB-77 of 1 × 10^−5^ g/mL, the LSPR *λ*_max_ was 714 nm and the peak position was red-shifted by 23 nm as compared with that of the hybrid Au–Ag hexagonal lattice of triangular PNAs without the modification of SH-β-CDs.

Those results prove that the hybrid Au–Ag hexagonal lattice of triangular PNAs with the modification of SH-β-CD have large sensing effect on PCB-77 because of the larger shift of LSPR *λ*_max_. When the hybrid Au–Ag PNAs with SH-β-CDs are illuminated under the incidence light, as the dielectric constant of the surrounding medium changes the effective refractive index of the medium will be changed. We believe the reason is that as the cyclodextrin is combined with the hybrid Au–Ag PNAs, the vibration of surface plasmon resonances will become slower. The resonance wave between nanoparticles will have a longer wavelength as the resonant frequency decreases and the LSPR *λ*_max_ is shifted to larger value. When the hybrid Au–Ag hexagonal lattice of triangular PNAs with the modification of SH-β-CD was used to sense the PCB-77, the PCB-77 will combine with SH-β-CD. Thus, the vibration units of entire arrays become larger, and the frequency of vibration becomes slower. The hybrid Au–Ag hexagonal lattice of triangular PNAs become more easily to absorb the wave with longer wavelength (or lower frequency), which is the reason leading to the increase of the red shift.

**Figure 12:**
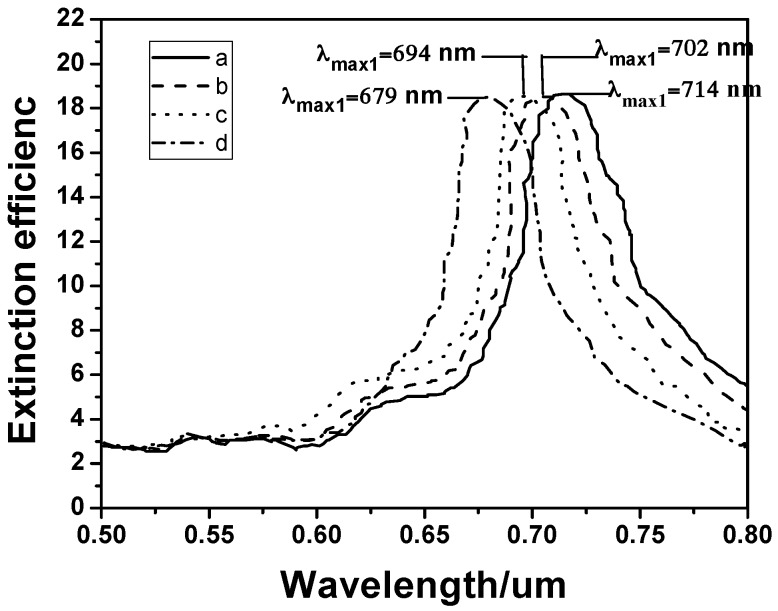
LSPR spectra of the SH-β-CDs-modified hybrid Au–Ag hexagonal lattice of triangular PNAs as a function of PCB-77 concentration: (**a**) 1 × 10^−5^ g/mL; (**b**) 1 × 10^−6^ g/mL; (**c**) 1 × 10^−7^ g/mL; and (**d**) 1 × 10^−8^ g/mL.

The SH-β-CDs-modified hybrid Au–Ag hexagonal lattice of triangular PNAs were placed in the PCB-77 solution with concentrations of 1 × 10^−5^ g/mL, 1 × 10^−6^ g/mL, 1 × 10^−7^ g/mL, and 1 × 10^−8^ g/mL, respectively, and the acquired absorption spectra are shown in Figure 12. We believe that the poor water solubility of PCB-77 promotes it to enter the high fat soluble cyclodextrin cavity area and combine with it firmly. When the concentration of PCB-77 decreased from 1 × 10^−5^ g/mL to 1 × 10^−8^ g/mL, the absorption spectrum had a blue shift of 50 nm. As the concentration of PCB-77 decreases, the volume of PCB-77 entering in the cyclodextrin cavity region decreases. The changes for the dielectric property and refractive index of the hybrid Au–Ag hexagonal lattice of triangular PNAs decrease, and that will lead to the continuing reduction of red shift. When the results in Figure 11 and Figure 12 are compared, the shift of the *λ*_max_ of LSPRs spectra is easily observed, even when concentration of PCB-77 is only 1 × 10^−8^ g/mL. The result suggests that the lowest detectable concentration for PCB 77 in the SH-β-CDs-modified hybrid Au–Ag hexagonal lattice of triangular PNAs is 1 × 10^−8^ g/mL. These results suggest, again, that the SH-β-CDs-modified hybrid Au–Ag PNAs can be investigated as high efficient sensor for the detection of polychlorinated biphenyls with low concentration.

**Figure 13:**
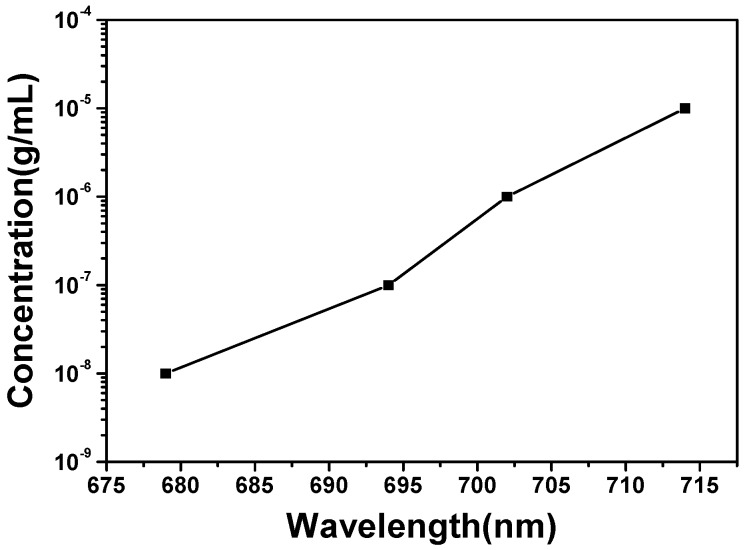
Calibration curve for the *λ*_max_ of LSPRs spectra and log (concentration) of PCB-77.

The calibration curve for the *λ*_max_ of LSPRs spectra and log (concentration) of PCB-77 are shown in Figure 13. As the figure shows, the *λ*_max_ of LSPRs spectra was linearly changed from the 714 nm to 679 nm as the concentration of PCB-77 was changed from 1 × 10^−5^ g/mL to 1 × 10^−8^ g/mL. Even when the concentration of PCB-77 was only 1 × 10^−8^ g/mL, the *λ*_max_ of LSPRs spectra has a 2 nm shift. The limit of detection (LOD) of this SPR sensor system was obtained using Equation (2) [39].

(2) $$Concentration_{LOD}=\frac{Comcentration\;of\;the\;analyte}{SPR\;response}*3S\text{tandard }deviation$$

The standard deviation (SD) of the signal was recorded over 100 s in the stable state. The SD of the blank measure of PCB-77 for SH-β-CDs-modified hybrid Au–Ag hexagonal lattice of triangular PNAs was 0.0036%. Therefore, the LOD of the concentration was calculated using Equation (2), and the respective value was 9.87 ng/mL. Thus, the SPR sensor can detect the PCB-77 at a low level of concentration. These results prove that the hybrid Au–Ag hexagonal lattice of triangular PNAs can be used as a sensor for polychlorinated biphenyl (PCB).

## 5. Conclusions

In this study, wavelengths with maximum extinction efficiency of LSPRs spectra were caused by the in-plane dipolar excitation. The wavelengths were 583 nm and 677 nm for hexagonal lattice of triangular and square lattice of quadrate hybrid Au–Ag nanoparticle arrays, respectively. The RIS values were 673 nm/RIU and 521 nm/RIU and the FOM values were 6.21 and 6.04 for the hexagonal lattice of triangular and square lattice of quadrate hybrid Au–Ag PNAs, respectively. The *λ*_max_ of LSPRs spectra of the hybrid Au–Ag hexagonal lattice of triangular PNAs was revealed at 671 nm. As the hybrid Au–Ag hexagonal lattice of triangular PNAs without and with the modification of SH-β-CD were used to measure the PCB-77 of 1 × 10^−5^ g/mL, the *λ*_max_ of LSPRs spectra were 691 nm and 714 nm, respectively. When the concentration of PCB-77 decreased from 1 × 10^−5^ g/mL to 1 × 10^−8^ g/mL, the *λ*_max_ of LSPRs spectra of the hybrid Au–Ag hexagonal lattice of triangular PNAs with the modification of SH-β-CD was shifted from 714 nm to 679 nm. The standard deviation (SD) of the signal was recorded over 100 s in the stable state. The SD of the blank measure of PCB-77 for SH-β-CDs-modified hybrid Au–Ag hexagonal lattice of triangular PNAs was 0.0036% and the LOD of the concentration was 9.87 ng/mL, respectively. However, the results in this study suggest that we can develop a highly efficient chemosensing technique for the detection of polychlorinated biphenyls by directly using the periodic nanoparticle arrays.

## Figures and Tables

**Table 1:** Refractive index sensitivity (RIS), width at half maximum (FWHM), and figure of merit (FOM) using for hybrid Au–Ag nanoparticle arrays under two different structures.

Feature/Characteristic	Hexagonal Array	Tetragonal Array
RIS (nm/RIU)	673	521
FWHM (nm)	108	86
FOM	6.21	6.04
